# A simple method for co-segregation analysis to evaluate the pathogenicity of unclassified variants; *BRCA1 *and *BRCA2 *as an example

**DOI:** 10.1186/1471-2407-9-211

**Published:** 2009-06-29

**Authors:** Leila Mohammadi, Maaike P Vreeswijk, Rogier Oldenburg, Ans van den Ouweland, Jan C Oosterwijk, Annemarie H van der Hout, Nicoline Hoogerbrugge, Marjolijn Ligtenberg, Margreet G Ausems, Rob B van der Luijt, Charlotte J Dommering, Johan J Gille, Senno Verhoef, Frans B Hogervorst, Theo A van Os, Encarna Gómez García, Marinus J Blok, Juul T Wijnen, Quinta Helmer, Peter Devilee, Christi J van Asperen, Hans C van Houwelingen

**Affiliations:** 1 Department of Medical Statistics and Bioinformatics, Leiden University Medical Center, Leiden, The Netherlands; 2 Center for Human and Clinical Genetics, Leiden University Medical Center, Leiden, The Netherlands; 3 Department of Pathology, Leiden University Medical Center, Leiden, The Netherlands; 4 Department of Clinical Genetics, Erasmus Medical Center, Rotterdam, The Netherlands; 5 Department of Genetics, University Medical Center, Groningen University, Groningen, The Netherlands; 6 Department of Human Genetics, Radboud University Nijmegen Medical Center, Nijmegen, The Netherlands; 7 Department of Medical Genetics, University Medical Center Utrecht, Utrecht, The Netherlands; 8 Department of Clinical Genetics, VU University Medical Center, Amsterdam, The Netherlands; 9 Family Cancer Clinic, Netherlands Cancer Institute, Antoni van Leeuwenhoek Hospital, Amsterdam, The Netherlands; 10 Department of Clinical Genetics, Academic Medical Center, Amsterdam, The Netherlands; 11 Department of Clinical Genetics, University Medical Center, Maastricht, The Netherlands; 12 Department of Genetics and Cell Biology, University Medical Center, Maastricht, The Netherlands

## Abstract

**Background:**

Assessment of the clinical significance of unclassified variants (UVs) identified in *BRCA1 *and *BRCA2 *is very important for genetic counselling. The analysis of co-segregation of the variant with the disease in families is a powerful tool for the classification of these variants. Statistical methods have been described in literature but these methods are not always easy to apply in a diagnostic setting.

**Methods:**

We have developed an easy to use method which calculates the likelihood ratio (LR) of an UV being deleterious, with penetrance as a function of age of onset, thereby avoiding the use of liability classes. The application of this algorithm is publicly available http://www.msbi.nl/cosegregation. It can easily be used in a diagnostic setting since it requires only information on gender, genotype, present age and/or age of onset for breast and/or ovarian cancer.

**Results:**

We have used the algorithm to calculate the likelihood ratio in favour of causality for 3 UVs in *BRCA1 *(p.M18T, p.S1655F and p.R1699Q) and 5 in *BRCA2 *(p.E462G p.Y2660D, p.R2784Q, p.R3052W and p.R3052Q). Likelihood ratios varied from 0.097 (*BRCA2*, p.E462G) to 230.69 (*BRCA2*, p.Y2660D). Typing distantly related individuals with extreme phenotypes (i.e. very early onset cancer or old healthy individuals) are most informative and give the strongest likelihood ratios for or against causality.

**Conclusion:**

Although co-segregation analysis on itself is in most cases insufficient to prove pathogenicity of an UV, this method simplifies the use of co-segregation as one of the key features in a multifactorial approach considerably.

## Background

High throughput technologies and more sensitive mutation detection systems in the DNA diagnostic laboratories have led to an increasing number of sequence variants in the major cancer-predisposing genes for which the clinical significance is unknown. In approximately 15% of the DNA mutation scannings of *BRCA1 *or *BRCA2 *in breast cancer families, the test result is difficult to interpret because an unclassified variant is found (UV, or 'variant of uncertain clinical significance' (VUCS) or 'variant of uncertain significance' (VUS) [1]. The risk for breast and ovarian cancer might be as high as for classical pathogenic mutations, but they might also be negligible. The decision for or against prophylactic surgery must in these cases be based entirely on the family cancer history, which never yields risks comparable to pathogenic mutations. In addition, predictive testing and preventive surgery are not offered to their healthy relatives, although these relatives may experience anxiety for carrying the UV [2].

A variety of approaches have been used to assess the clinical relevance of these UVs [3-9]. These include 1) *in silico *predictions based on evolutionary conservation, position and nature of the amino acid change, 2) functional analysis of the variant using *in vitro *assays directed at specific functional domains of the protein, 3) population genetics analyzing frequency of the variant in cases and controls and co-occurrence of the UV with a known deleterious mutation in one or more tested individuals and 4) clinical validation using family history, co-segregation of the variant with the disease in pedigrees as well as relevant features of *BRCA1*- and *BRCA2*-associated tumours.

A functional assay would ideally be capable of distinguishing pathogenic from neutral variants. However, although these assays are available, their results are very difficult to interpret clinically and can only be used for a subset of unclassified variants located in specific domains of the genes. This is because *BRCA1 *and *BRCA2 *are multifunctional proteins and these assays will generally only interrogate one of those functions. Since none of the approaches mentioned above have by itself been able to provide compelling evidence for or against pathogenicity of UVs, multifactorial likelihood models integrating these features have been developed [3,4]. As shown by Goldgar *et al*., the significance of the analysis was highly dependent on co-occurrence and co-segregation data. Recently, the genetic evidence for or against disease causality was reported for a large number of variants with the use of a large database of tested individuals collected analyzing co-occurrence, personal and family history of cancer as well as co-segregation with disease, the latter only in a subset of probands [5]. Although co-occurrence has shown to be a powerful tool to obtain high odds in favour of neutrality, this method requires large databases of tested individuals, such as collected by Myriad Genetics laboratories, and these data are not publicly available. In addition, family-history analysis has demonstrated to be very useful although it might be susceptible to bias due to population-specific ascertainment. Co-segregation (occurrence of UVs in affected individuals) is not affected by selection bias because it considers the distribution of the genotypes given the phenotypes and the genotype of the proband (the first person that was genotyped in the family and carried a UV). Co-segregation analysis is therefore regarded as a robust approach since it directly relates to the disease risk whereas the absence of co-segregation provides strong evidence against pathogenicity.

In order to classify UVs via co-segregation, a simple Bayesian method to assess causality of rare sequence variants was provided by Petersen *et al*. [10]. In contrast to Petersen, Thompson *et al*. [11] have developed a more general method based on the full pedigree likelihood. All available genotype information from the family is used, including any unaffected individuals who have been tested. The two methods either use a defined penetrance in carriers *versus *non-carriers and ignore the age of onset [11], or specify liability classes which define the age range of family members in intervals for which the penetrance is supposed to be constant [10]. Using the full age of onset information however will result in more reliable estimates of the likelihood ratio (LR).

We have developed an algorithm to calculate the likelihood ratio of a UV being deleterious based on co-segregation analysis, using the precise age of onset information. The method can easily be used in a diagnostic setting since it requires only information on gender, genotype, present age and/or age of onset for breast and/or ovarian cancer.

## Methods

In collaboration with the DNA diagnostic laboratories of the Clinical Genetic Centres in the Netherlands, 8 UVs have been selected for this study (Table 1, detailed description can be found in Additional file 1; Table S1). The Human Genetic Variation Society (HGVS) approved guidelines http://www.hgvs.org/mutnomen have been used for *BRCA1 *and *BRCA2 *nomenclature [12]. To facilitate published data comparison, also the traditional nomenclature is listed (Breast Cancer Information Core, http://research.nhgri.nih.gov/bic/). GenBank accession no. NM_007294.2/NP_009226.1 and NM_000059.3/NP_000050.1 have been used for *BRCA1 *and *BRCA2 *mRNA and protein numbering respectively.

**Table 1:** *BRCA1 *and *BRCA2 *missense variants analysed and likelihood ratio in favor of causality

Gene	**HGVS**^1^	**traditional**^2^	protein	**# fam**^3^	**LR per family**^4^	**LR-variant**^5^
*BRCA1*	c.53T>C	172T>C	p.M18T	2	5.7004(2^+^)	7.9777
					1.3995(4^+^; 6_+_; 3_-_)	
*BRCA1*	c.4964C>T	5083C>T	p.S1655F	1	6.7425(3^+^; 2_+_)	6.7425
*BRCA1*	c.5096G>A	5215G>A	p.R1699Q	1	1.4280(2^+^)	1.4280
*BRCA2*	c.1385A>G	1613A>G	p.E462G	4	0.7738(1^+^; 1_+_)	0.0965
					0.6757(1^+^; 1_+_)	
					1.2459(1_+_; 1_-_)	
					0.1481(1^+^; 1^-^)	
*BRCA2*	c.7978T>G	8206T>G	p.Y2660D	3	1.8579(2^+^),	230.6927
					11.1565(2^+^)	
					11.1297(2^+^; 3_+_)	
*BRCA2*	c.8351C>T	8579G>A	p.R2784Q	1	1.8101(1^+^; 1_+_)	1.8101
*BRCA2*	c.9154C>T	9382C>T	p.R3052W	2	3.0651(2^+^),	12.1960
					3.9790(3^+^)	
*BRCA2*	c.9155G>A	9383G>A	p.R3052Q	1	0.2208(1^+^; 1^-^)	0.2208

^1^The Human Genetic Variation Society (HGVS) approved guidelines http://www.hgvs.org/mutnomen have been used for *BRCA1 *and *BRCA2 *nomenclature [12].

^2^To facilitate published data comparison, also the traditional nomenclature is listed (Breast Cancer Information Core, http://research.nhgri.nih.gov/bic/). GenBank accession no. NM_007294.2/NP_009226.1 and NM_000059.3/NP_000050.1 have been used for *BRCA1 *and *BRCA2 *mRNA and protein numbering respectively.

^3^Number of families with at least two genotyped family members.

^4^Likelihood ratio per family. Between brackets the number of affected individuals carrying the UV (n^+^) or unaffected individuals with the UV (n_+_), affected individuals without the UV (n^-^) or unaffected individuals without the UV (n_-_).

^5^The overall likelihood ratio is derived by the product of the LR over the independent families.

Each laboratory selected 5 UVs in either *BRCA1 *or *BRCA2 *which were of particular interest for them (e.g. multiple families with same UV, large families in which the UV was segregating). A long-list was made which contained all the selected UVs (14 *BRCA1*, 17 *BRCA2*). From this list, UVs were selected that met a least one of the following criteria: 1) Grantham score above 100, 2) The UV is located in a functional domain [13], 3) The amino acid has a high degree of evolutionary conservation (i.e. also in non-mammalian species). In addition, priority was given to those UVs found in more than one family and/or genetic centre. UVs co-occurring with a pathogenic mutation in the same gene (data from Netherlands) in the proband were excluded. 15 Dutch families, in which one of the 8 selected UVs had been found and for which at least two family members were genotyped were included in our study. Some of the UV's were detected in more than one family. Families were tested for *BRCA1*/2 after genetic counselling of clinically presumed hereditary breast and/or ovarian cancer, when the mutation detection rate is above 10% [14] (e.g., two breast cancer cases with one case under age 50, more than 3 first-degree relatives in two successive generations with breast cancer under age 60, breast and/or ovarian cancer families), or if breast cancer was diagnosed at a relatively young age (i.e., one patient younger than 35 years old). The proband is defined as the first person in the family who is genotyped and tested positive for one of the selected UVs. The pathogenic control group consisted of families in which a pathogenic mutation was detected in *BRCA1 *or *BRCA2 *(Table 2). The neutral control group consisted of families in which genetic variants were found that are considered neutral variants with respect to breast and/or ovarian cancer risk (Table 3) [3,15].

**Table 2:** Likelihood ratio in favor of causality for selected pathogenic mutations

Gene	**HGVS**^1^	**traditional**^2^	protein	**# fam**^3^	**LR per family**^4^	**LR-variant**^5^
*BRCA1*	c.81-6T>A	IVS2-6T>A	p.Cys27fsX1	1	6.4287(5^+^; 3_+_; 1^-^; 3_-_)	6.4287
*BRCA1*	c.213-12A>G	IVS5-12A>G	p.Arg71fsX20	1	10.2701(1^+^; 3_+_; 6_-_)	10.2701
*BRCA1*	c.1292dup	1411insT	p.Leu431PhefsX5	2	1.7312(2^+^; 1_+_; 1_-_)	3.3715
					1.9475(1^+^; 2_+_; 1_-_)	
*BRCA1*	c.2193_2197del	2312delAGAAG	p.Glu733ThrfsX5	1	4.7089(2^+^; 1_+_; 4_-_)	4.7089
*BRCA1*	c.5095C>T	5214C>T	p.Arg1699Trp	3	22.8602(3^+^; 6_+_; 2^-^; 4_-_)	402.164
					2.2720(1^+^; 1_+_; 1_-_)	
					7.7431(2^+^; 1_+_)	
*BRCA2*	c.2806_2809del	3034delAAAC	p.Ala938ProfsX21	1	1.9901(2^+^; 1_+_; 1_-_)	1.9901
*BRCA2*	c.3269del	3497delT	p.Met1090SerfsX14	1^6^	87.46(5^+^)	87.46
*BRCA2*	c.3599_3560del	3827delTG	p.Cys1200X	1	1.1876(1^+^; 3_+_; 1_-_)	1.1876
*BRCA2*	c.8067T>A	8295T>A	p.Cys2689X	1	12.2424(3^+^; 1_+_; 4_-_)	12.2424
*BRCA2*	c.8773C>T	9001C>T	p.Gln2925X	1	1.8827(1^+^; 3_+_)	1.8827

^1–5 ^For clarification see Table 1.

^6 ^Pedigree is shown in Figure 2.

**Table 3:** Likelihood ratio in favor of causality for selected neutral variants

Gene	**HGVS**^1^	**traditional**^2^	protein	**# fam**^3^	**LR per family**^4^	**LR-variant**^5^
*BRCA1*	c.135-15_135-12del^6^	IVS4-15delCTTT	p.=	1^9^	3.8592 (2^+^)	3.8592
*BRCA1*	c.2613G>A^7^	2732G>A	p.Pro871Pro	1	1.8533(2^+^)	1.8533
*BRCA1*	c.5152+20T>A^7^	IVS18+20T>A	p.=	1	3.6711(1^+^; 1_+_)	3.6711
*BRCA2*	c.125A>G^8^	353A>G	p.Y42C	2	0.9728(2_+_)	0.7405
					0.7612(1^+^; 4_+_)	

^1–5 ^For clarification see Table 1.

These variants are regarded to be neutral towards cancer risk based on ^6^Vreeswijk *et al*. [15], ^7^Splice site prediction analysis and ^8^Goldgar *et al*. [3].

^9 ^Pedigree is shown in Additional file 1; Figure S4.

### Likelihood ratio using co-segregation data

In this subsection, we derive the relevant likelihood ratio to evaluate the evidence for the causality associated with the UV referred to breast or ovarian cancer. The first assumption is that the proband carries a UV in *BRCA1 *or *BRCA2*.

The notation G stands for the genotype of the UV in the family. Hence, G = 1 if the UV is present and G = 0 if not.

The genotype is observed in at least one other family member, but certainly not in all. Phenotypic information is available in most family members and denoted by *P*. Pedigrees are pruned to leave out non-informative branches (i.e. no cancer and no genotype information).

We use the notations "d", "n", "o", "u", "f", "p" for "deleterious", "neutral", "observed", "unobserved", "family" and "proband" respectively. The likelihood ratio for pathogenicity based on segregation within families is then equal to

By definition of "neutral"

One has

Hence,

We have to take into account all family members with unobserved (missing) genotypic information. If we do so, we obtain

So, the building blocks are *P*(*G*_*f*_|*G*_*P *_= 1) and *P*_*d*_(*P*_*f*_|*G*_*f*_).

We can now make some simplifying assumptions

a. the mutation is very rare. That helps enormously in enumerating all possible genotypes in the family and computing *P*(*G*_*f*_|*G*_*P *_= 1)

b. if the mutation is deleterious and known to be present in the family, the genotype for that mutation is the dominant factor in computing *P*_*d*_(*P*_*f*_|*G*_*f*_), that is to say that the probability of the phenotype for an individual only depends on her/his genotype and not on the phenotypes of the other family members. This implies that , where *i *changes over all family members.

If the mutation is deleterious, we assume that the penetrance of the mutation (defined by the probability of acquiring the disease before age *t*) is given by the known penetrance for *BRCA1 *or *BRCA2*, respectively. We apply the penetrances as in Jonker *et al*. [16], the smoothed versions of the known penetrances based on normal distribution functions. We denote the density and cumulative functions of normal distribution by *φ *and Φ respectively. The general form is given by *F*(*t*|*r*, *μ*, *σ*) = *r *Φ((*t *- *μ*)/*σ*). Values of *r *(life time risk), *μ *(age of diagnosis) and *σ *(standard deviation) for the breast and ovarian cancer are given in Additional file 1; Table S2. The penetrance functions are plotted in Additional file 1; Figure S1. The phenotypic likelihood is given by 1 - *F*(*t*|*r*, *μ*, *σ*) if an individual is disease free at age *t*, and by the derivative *f*(*t*|*r*, *μ*, *σ*) = *rφ*(*t *- *μ*)/*σ*)/*σ *if the individual has cancer at age *t*. Breast and ovarian cancer are assumed to be statistically independent given the genotype of the deleterious mutation.

The likelihood for two sided breast cancer cases at onset ages *t*_1 _and *t*_2 _(≥ *t*_1_) is given as the product of the derivatives of  and [17].

For a specific variant, if there are multiple families carrying the variant, the overall likelihood is derived by the product of the likelihood ratios over the independent families, assuming that the specific variant is not in linkage disequilibrium with an unknown deleterious mutation.

### An algorithm to enumerate possible genotypes

To analyze co-segregation and to compute *P*(*G*_*f*_|*G*_*P *_= 1) under the rare genotype assumption, one has to explore all the different possibilities of the genotypes of the family members. The genotype takes values 1 for the carriers of the variant and 0 for non-carriers. In our data set the proband is by definition carrier of a variant (genotype 1).

**Figure 1:**
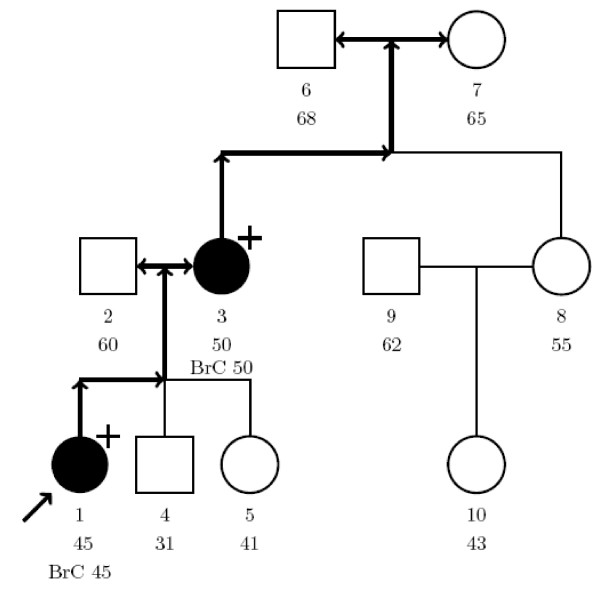
**Illustration of our model**. Hypothetical pedigree for the illustration of our model. Individuals are numbered 1–10 for identification and underneath the age at last contact is listed. Open circle: female, open square: male, closed circle: affected with breast (or ovarian) cancer at age × (BrCx). Unless specified by + (carrier of UV), - (no carrier), individuals are not genotyped. Proband is indicated by arrow.

The algorithm that is at the basis of our computational algorithm is demonstrated using the pedigree of Figure 1.

The members of the pedigree are numbered 1,...,*n*. In our example, the proband is given number 1. A genotypic configuration is described by a vector G = (*G*_1_,...,*G*_*n*_) of 0's and 1's, *G*_*i *_= 1 if individual *i *carries the UV and *G*_*i *_= 0 if it is a non-carrier. Under the rare mutation assumption and the selection on the proband, all the possibilities have to meet the following

1. *G*_*P *_= 1.

2. If two parents have *G *= 0, all children have *G*_*i *_= 0.

3. If for an individual *G*_*i *_= 1, one (and only one) of his/her parents must have the mutation, i.e. *G *= 1.

4. Exactly one of the founders has *G *= 1.

Conditions 3 and 4 are equivalent formulations of the rare mutation assumption.

In order to construct an enumeration of all possible genotypic configurations, we proceed as follows

#### Step 1

Finding possible founders

Going upwards from the proband we can find all founders that can be reached from the proband. Given a founder, all family members in the path from the founder to proband must have the mutation as well. All the pedigree members that do not descend from the founder with the mutation cannot have the mutation.

In the example (Figure 1), person 1 is the proband carrying the UV. There are three possible founders: person 2, 6 or 7. We will discuss these three possibilities below.

1) If person 2 carries the mutation, then numbers 3, 6, 7, 8 and 10 can not have the mutation. Person 4 and 5 may have the mutation. We first indicate the genotype of numbers 4 and 5 by *x *and then obtain all possibilities for *x'*s. So all the possibilities in case that number 2 carries the mutation are contained in the following vector where the upper line represents the number of the person in the pedigree and the lower line represents the genotype information.

2) If number 7 has the mutation, then numbers 2 and 6 cannot have the mutation. Number 3 will carry the mutation and numbers 4, 5, 8 and 10 may have the mutation. Again we first indicate the genotype of numbers 4, 5, 8 and 10 by *x *and then obtain all possibilities for *x*'s. So all the possibilities for the case that number 7 has the mutation are contained in the following vector

3) If number 6 has the mutation, then we repeat the situation 2) above with numbers 6 and 7 replaced by each other.

Hence, all the possible configurations are contained in the vectors *M*_1 _and *M*_2_.

The situations 1), 2) and 3) are also shown in Additional file 1; Figure S2A. This figure might be useful to understand how the algorithm is built.

The *x'*s have to be filled to obtain the enumeration. To do this in an efficient way we have to determine the "generation" of the *x*'s.

#### Step 2

Setting the generations.

We use the following iterative algorithm to define the "generation" *H *for the

"open" genotypes:

a. if both parents have known genotype, then *H *= 2,

b. if one parent has "open" genotype (two is impossible), then *H*_*i *_= *H*_*parent*_+1.

We fill in the generations at the "open" genotypes. We obtain

#### Step 3

Filling the "hole"

The general principle is that an open genotype must be "0" if both parents have *G*_*i *_= 0 and can be 0 or 1 if one of the parents has *G*_*i *_= 1. That leads to the filling and splitting algorithm shown in Additional file 1; Table S3.

Additional file 1; Figure S2B shows the situation 1) above. As mentioned above, all possibilities in this situation are contained in vector *M*_1_.

We start with this vector and we need to find all different possibilities for the 2's in this vector. We start with the member number 4.

Because one of the parents has the mutation, number 4 can have *G *= 0 and *G *= 1. For each of these possibilities, (with the same reason) number 5 also has two possibilities *G *= 0 and *G *= 1. In fact when a parent has *G *= 1, there is a split, otherwise there is no split.

The situation 2) above is shown in Additional file 1; Figure S3. As mentioned above, all possibilities in this situation are contained in vector *M*_2_.

We start with this vector and we need to find all different possibilities for the 2's and 3's in this vector. We start with the member number 4. As our explanation for Additional file 1; Figure S2B, we have a split for number 4. For each of the possibilities, we have another split for number 5 and for each of them; we have another split for number 8. Then all the 2's are filled and we arrive at the 3 for number 10. Here we have a split just for the possibilities that number 8 has G = 1 otherwise there is no splits and number 10 has G = 0.

Using our algorithm, the two matrixes *M*_1 _and *M*_2 _become

To compute *P*(*P*_*f*_|*G*_*p *_= 1) one needs to derive the probability of each possibility.

Note that

and

So, each split halves the probability. This is shown in Additional file 1; Figure S2.

The probability of each possibility is the product of the 1/2's. In fact a split occurs when a member of the family has a parent with *G *= 1. Hence, the probability of a possibility is 1/2 to the power of the number of family members who have one parent with *G = 1 *in the possibility.

For the two matrixes, *M*_1 _and *M*_2_, we obtain the probabilities below for the respective rows.

Assuming that the proband in Figure 1 carries a *BRCA1 *mutation, the likelihood ratio in favour of causality is 1.66. The likelihood ratio becomes 1.76 in case of a *BRCA2 *mutation, due to differences in penetrance between both genes.

The algorithm can be run on the website at http://www.msbi.nl/cosegregation. On the website, users can upload a file containing the pedigree information and the application will calculate the likelihood ratio instantly. A detailed instruction for the use of the method is available as Additional file 2 as well as on the website.

## Results

We have calculated the likelihood ratios for 8 UVs in *BRCA1 *and *BRCA2 *using the method described in the Methods section. In Table 1 the likelihood ratios are given for the separate UV-families, as well as the combined odds for an UV for which multiple families were available. Likelihood ratios for families with pathogenic mutations and neutral variants are summarized in Table 2 and 3 respectively.

The multifactorial likelihood model described by Goldgar *et al*. [3], considered UVs with overall odds of causality >1000:1 (LR>1000) as pathogenic and those with odds of causality <1:100 (LR<0.01) as neutral.

Except for the *BRCA2 *p.Y2660D variant, with a LR in favour of causality of more than 230, none of the UV's has a very outspoken LR in favour of causality (LR>1) or neutrality (LR<1). Moreover, there is quite some heterogeneity between families. Similarly, we see that the evidence for the pathogenic and neutral controls is not very strong either. In this section we describe some further data-analysis to get a better insight into the usefulness of co-segregation in the classification of UV's. Moreover, we want to explore what the best genotyping strategy should be: Should we genotype close or distant relatives of the proband and patients or healthy individuals?

**Figure 2:**
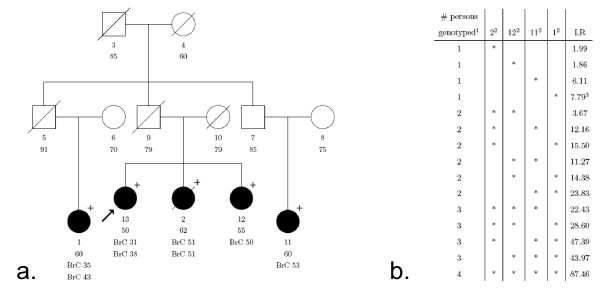
**Likelihood ratio in favour of causality for different genotyping patterns**. a. Pedigree with *BRCA2 *mutation c.3269del. Individuals are numbered 1–13 for identification and underneath the age at last contact is listed. Open circle: female, open square: male, /: deceased, closed circle: affected with breast (or ovarian) cancer at age × (BrCx). In case of bilateral breast cancer this is listed below the first occurrence. Unless specified by + (carrier of UV), - (no carrier), individuals are not genotyped. Proband is indicated by arrow. b. Likelihood ratios in favour of causality if only a subset of the family members are genotyped and in different combinations. ^1^The number of family members that is genotyped positive for the mutation, in addition to the proband, individual 13, who carries the *BRCA2 *mutation c.3269del mutation. ^2^Family member number in pedigree. ^3^Likelihood ratio in favour of causality when individual 13 and 1 carry the mutation.

To explore the latter issue we study a pedigree with a pathogenic *BRCA2 *mutation (c.3269del with a LR of 87.46) in more detail (Figure 2). We calculated what the LR would be if we had genotyped only a subset of the family members and in different combinations. Typing for example one sister of the proband with breast cancer at age 50 (individual 12) will give a LR of 1.86 when she carries the mutation, whereas a positive genotype (i.e. carrier of the UV) of a third grade related cousin with breast cancer at age 53 (individual 11) results in a LR of 6.11. Two affected sisters with the UV or two affected cousins will generate a LR of 3.67 and 23.83 respectively. From this analysis it is clear that typing distantly related individuals with extreme phenotypes (i.e. very early onset cancer or old healthy individuals) is most informative and gives the strongest likelihood ratios for or against causality. Small pedigrees with only one or two affected individuals do not carry very much information that helps to decide on the clinical significance of the UV. Generally speaking it is helpful to genotype as many individuals as possible. If it is only possible to type a limited number of individuals, the advice is to type individuals with "extreme phenotypes" in these breast cancer families. Distant affected young relatives which are carriers and nearby unaffected old non carriers (>60 years of age) are a very strong indication for causality of the variant whereas variants found in distant unaffected old family members and not in nearby affected young members will very likely be neutral to cancer risk.

To explore the sensitivity of the method we have to look into one of the pedigrees with a neutral variant (*BRCA1 *c.135-15_135-12del; Additional file 1; Figure S4). We distributed the rare variant randomly from the proband onwards using the algorithm described in the Methods section and looked what distribution of the LR we would get depending on who we genotyped and what the outcome would be. If we would type everybody, we would obtain a distribution of the Likelihood Ratio for which the probability of getting a LR>1 is almost 20% and the probability of getting LR>3 almost 8%. The probability of obtaining a LR<0.1 in favour of neutrality, is also almost 8% (Additional file 1; Figure S5).

One might wonder if it would not have been wiser to genotype another pedigree member. However, a more detailed analysis of the simulation data (not shown) reveals that the LR of 2.97 that we get if we only type individual 4 is no indication that the wrong individual was genotyped. Generally, individuals that show strong evidence when their genotype is positive also show strong evidence when the genotype is negative.

## Discussion

To assess the clinical relevance of unclassified variants, a number of approaches have been described and the analysis of segregation of the variant in affected family members has been shown to be very powerful [3]. Co-segregation analysis is usually done with statistical software for linkage analysis in pedigrees [10,11] but a major disadvantage of the currently used methods is that they are not user-friendly and ignore the precise age of onset of the disease since either liability classes or a constant penetrance in carriers is used. We have therefore developed an easy to use method which calculates the likelihood ratio with penetrance as a function of age of onset. The analysis requires at least two genotyped persons with information on gender and present age or age onset for breast and/or ovarian cancer. The software we developed has some similarity with the well-known BRCAPRO software and requires similar input [17]. It is written in MATLAB and the application can be run on our website: http://www.msbi.nl/cosegregation

### Essential assumptions

One of the critical steps in our analysis is the selection of the proband. In most cases, this will be the youngest affected family member that was tested positive for the UV. The algorithm analyses the segregation of the UV from the proband and the results can differ according to which person is used as proband. In principle it is possible to allow more complex genotypic ascertainment schemes, but in practice it is essential to know who the proband is.

Another important assumption is that "causal" UV's show the same penetrance as the known deleterious mutations in *BRCA1 *and *BRCA2*. In our analysis we did not allow for penetrance functions that depend on the particular mutation although it would be easy to redo the calculations under the assumption of mutation-specific penetrance. Since it would reduce the power of our method, such an analysis would only be feasible in large pedigrees with many genotyped individuals. Thirdly, we assume that the UV is not in linkage disequilibrium with an unknown deleterious mutation. Finally, we computed UV specific LR's by simply multiplying the LR's of the families with the same UV under the assumption of homogeneity of the effect. Unfortunately, we do not have data on enough families with the same UV to analyze the heterogeneity properly.

### The algorithm

The algorithm works by complete enumeration of all genotypic configurations under the assumption that the variant is rare. This method is very fast if the pedigree is not too big. In practice we prune non-informative branches by hand before applying the algorithm. An alternative for manual pruning is to combine our complete enumeration with the peeling algorithm of Elston- Stewart [18]. Such an algorithm would retain all information, would be able to handle large pedigrees and would speed up the computations. We use the algorithm for breast cancer related to *BRCA1 *and *BRCA2 *mutations, but it can be easily applied to other cancers, provided the penetrance functions of the genes involved are known.

### Information per pedigree

As we pointed out in the results section, the amount of available information varies between pedigrees due to the size and complexity of the pedigree, and within pedigrees due to the variation in phenotype. As in linkage analysis [19], it pays off to select "extreme" individuals for genotyping in those breast cancer families. In our analysis individuals can have an extreme phenotype (very early onset of disease or disease free at old age) and an extreme genotype (distant relatives that are carrier or non-carriers near the proband). Evidence for causality is obtained if phenotype and genotype are concordant (e.g. carriers with early onset disease and non-carriers healthy family members at old age); evidence for neutrality is obtained if they are discordant (e.g. non-carriers with early disease or healthy old age carriers). As shown in the results section, it is straightforward to compute/simulate the LR's that can be obtained if certain individuals are genotyped. Such a simulation analysis can help in deciding whether to genotype at all, and, if so, who to genotype among those who are willing to be genotyped.

### Clinical findings

We have used the algorithm to calculate the likelihood ratio in favour of causality for 3 UVs in *BRCA1 *(i.e. p.M18T, p.S1655F and p.R1699Q) and 5 in *BRCA2 *(i.e. p.E462G p.Y2660D, p.R2784Q, p.R3052W and p.R3052Q). Likelihood ratios varied from 0.097 (*BRCA2*, p.E462G) to 230.69 (*BRCA2*, p.Y2660D). Co-segregation analysis on itself was therefore not powerful enough to classify any of these variants, based on the LR threshold for causality of 1,000:1 (LR>1000) and for neutrality of 100:1(LR<0.01) [3].

Previous studies have suggested that the *BRCA1 *p.S1655F variant and the p.R3052W in *BRCA2 *are likely to be deleterious (Additional file 1; Table S1). Our LRs of 6.74 and 12.20 respectively are in line with these findings and support the pathogenic nature of these variants.

The p.E462G (LR 0.097) and p.R3052Q (LR 0.22) variants in *BRCA2 *have odds below 1, suggestive for being neutral as has been described by others (Additional file 1; Table S1).

For the p.M18T and p.R1699Q variants in *BRCA1*, LRs of 7.98 and 1.43 respectively were not informative enough to classify them, in line with the inconclusive results obtained by other studies (Additional file 1; Table S1). These might be variants associated with an intermediate risk and depending on the type of analysis, the variants are classified as likely to be deleterious or neutral. Extensive co-segregation analysis in multiple families with these variants using different penetrance assumptions however might be sufficient to classify these variants in the future.

To date, no other information is available on the pathogenicity of the p.Y2660D and p.R2784Q variants in *BRCA2*. We have analysed co-segregation in 3 families with a p.Y2660D variant and found a LR of 230.69 which is highly suggestive for being a deleterious variant. More families have to be analysed before a definitive conclusion regarding the pathogenicity of this variant can be obtained.

A major problem with the use of co-segregation analysis is that extensive co-segregation analysis is rarely performed in these families, as a result of which only a few families could be used for our analysis. Since the cancer risk associated with these variants is unknown, the information that additional family members carry the variant will not change the screening advice. Therefore, clinical geneticist might be reluctant to perform additional DNA screening in these families, since the knowledge that someone carries the variant might cause anxiety although it is not established that it is associated with elevated cancer risk [20]. The main reason to perform DNA mutation screening in UV families should be however, the contribution to the assessment of the pathogenicity of the variant. We have used families in which either neutral or pathogenic *BRCA1 *or *BRCA2 *mutations segregate, to determine which family members should be typed to give the most informative results. By careful selection of family members to be typed, i.e. early breast cancer cases distantly related to the proband and old healthy individuals nearby, segregation analysis will be a powerful tool to assess the clinical significance of unclassified variants in *BRCA1 *and *BRCA2*.

Although functional data might be useful for classification, to date only functional assays are available for a subset of the UVs, namely those that are located in specific domains in the genes (e.g. BRCT domains in *BRCA1*) or those that affect splicing. Furthermore, the results of the functional assays are difficult to quantify and do not necessary reflect the influence on cancer risk, since *BRCA1 *and *BRCA2 *are multifunctional. The determination of the clinical significance of UVs should therefore preferably rely on clinical data, such as co-segregation analysis, which is directly related to disease risk and requires few assumptions. A disadvantage might be that most variants are very rare and that large international collaborations will be required to obtain enough informative families with the same UV to obtain LR>1000, the threshold for causality. There is one other drawback to the use of co-segregation analysis; the UV might be in linkage disequilibrium with an unknown deleterious mutation in *BRCA1 *or *BRCA2*. To reduced this possibility it is preferred that multiple families are included in the co-segregation analysis.

In the case of most unclassified variants, classification will depend on several data sources (i.e. co-occurrence, family history, tumour information, etc.) as have been described by others [3,4]. In line with the approach of Osorio *et al*. [6], we would like to opt for a model which is based on information that is readily accessible in a clinical setting. Clinical observations, such as co-segregation, family history and tumour information (e.g. LOH and CGH analysis), are directly related to cancer risk and relatively straightforward to quantify. Such a model will considerably increase the clinical utility of *BRCA1 *and *BRCA2 *genetic testing.

Since unclassified variants are also detected in genes underlying other genetic diseases, e.g. hereditary colon cancer, such a model might be applicable more broadly in a clinical genetic setting.

## Conclusion

Co-segregation analysis on itself is in most cases insufficient to prove pathogenicity of an UV. The presented method, with its user-friendly web application, simplifies the use of co-segregation as one of the key features in a multifactorial approach considerably. Users can upload a file containing the pedigree information on the website to calculate the likelihood ratio. Genotypes of distantly related individuals with extreme phenotypes (i.e. very early onset cancer or old healthy individuals) are most informative and give the strongest likelihood ratios for or against causality.

## Competing interests

The authors declare that they have no competing interests.

## Authors' contributions

LM designed the algorithm, performed the statistical analysis and drafted the manuscript. MPV conceived of the study, obtained financial support, coordinated the data acquisition, analyzed and interpreted data and authored the final version of the manuscript. RO, AO, JCO, AHH, NH, ML, MGA, RBL, CJD, JJG, SV, FBH, TAO, EGG, MJB and JTW were responsible for the selection of the UVs, DNA sequencing and the clinical data collection. QH was responsible for the web application of the algorithm. PD participated in the design of the study, obtained financial support and was involved in revision of the final version of the text. CJA participated in the design of the study, obtained financial support, was responsible for data acquisition, analyzed and interpreted data and authored the final version of the manuscript. HCH contributed to the concept and design of the study, supervised the development of the algorithm and helped draft the manuscript. All authors read and approved the final manuscript.

## Pre-publication history

The pre-publication history for this paper can be accessed here:

http://www.biomedcentral.com/1471-2407/9/211/prepub

## Supplementary Material

Additional file 1 **Additional Tables and Figures Mohammadi**. This file contains additional tables 1–3 and additional figures 1–5. Click here for file

Additional file 2 **Instruction method Mohammadi**. This document contains a description on the use of the co-segregation analysis website with detailed instruction on the preparation of the input file that contains the pedigree information. Click here for file
