# Supplementary material for: A simple method for co-segregation analysis to evaluate the pathogenicity of unclassified variants; BRCA1 and BRCA2 as an example
Source: BMC Cancer. 2009 Jun 29;9:211. doi: 10.1186/1471-2407-9-211 (PMC2714556; doi:10.1186/1471-2407-9-211)
Supplement: Additional file 1 — Additional Tables and Figures Mohammadi. This file contains additional tables 1–3 and additional figures 1–5. [file 1471-2407-9-211-S1.pdf]

Additional file 1

Tables and figures Mohammadi et al.

Table 1

							Align GVDV[6]				
Gene	HGVS[1]	traditional[2]	protein	# families[3]	BIC entries[4]	GS[5]	Conserved mammals/other	GV	GD	Prediction	Classification based on literature[7]
BRCA1	c.53T>C	172T>C	p.M18T	2	3	81	Y / N	14.30	81.04	Class C45	Inconclusive
BRCA1	c.4964C>T	5083C>T	p.S1655F	1	3	155	Y / N	57.75	102.86	Class C25	Predicted deleterious
BRCA1	c.5096G>A	5215G>A	p.R1699Q	1	11	43	Y / Y	0.00	42.81	Class C35	Inconclusive
BRCA2	c.1385A>G	1613A>G	p.E462G	4	35	98	Y / N	353.86	0,00	Class C0	Neutral
BRCA2	c.7978T>G	8206T>G	p.Y2660D	3	2	160	Y / Y	0.00	159.94	Class C65	No Information
BRCA2	c.8351C>T	8579G>A	p.R2784Q	1	4	43	Y / Y	0.00	42.81	Class C35	No Information
BRCA2	c.9154C>T	9382C>T	p.R3052W	2	8	101	Y / Y	0.00	101.29	Class C65	Predicted deleterious
BRCA2	c.9155G>A	9383G>A	p.R3052Q	1	3	43	Y / Y	0.00	42.81	Class C35	Neutral

Table 1

Description of the *BRCA1* and *BRCA2* missense variants in this study
[1]The Human Genetic Variation Society (HGVS) approved guidelines (ww.hgvs.org/mutnomen) have been used for *BRCA1* and *BRCA2* nomenclature (den Dunnen and Antonarakis, 2000). To facilitate published data comparison, also the traditional nomenclature is listed ([2]Breast Cancer Information Core, http://research.nhgri.nih.gov/bic/). GenBank accession no. NM_007294.2/NP_009226.1 and NM_000059.3/ NP_000050.1 have been used for *BRCA1* and *BRCA2* mRNA and protein numbering respectively.
[3]Number of families with at least two genotyped family members.
[4]BIC entries: number of times variant has been reported to BIC database (http://research.nhgri.nih.gov/bic; May 2008)
[5]GS: Grantham Score (Grantham, Science 185: 862-864 (1974).
[6]Sequences were used from mammalian* and non-mammalian species (reference sequences) as available on the Align GVDV website (http://agvgd.iarc.fr/alignments.php GVGD update 2007/11/08) (Tavtigian et al., 2005).
BRCA1: Homo sapiens* (NP_009225), Pan troglodytes* (Q9GKK8), Gorilla gorilla* (Q6J6I8), Pongo pygmaeus* (Q6J6J0), Macaca mulatta* (Q6J6I9), Mus musculus* (NP_033894), Canis lupus familiaris* (NP_001013434), Bos Taurus* (NP_848668), Monodelphis domesticus* (AAX92675), Gallus gallus (NP_989500), Xenopus laevis (AAL13037), Tetraodon nigroviridis (AAR89523), Strongylocentrotus purpuratus (EF152287).
BRCA2: Homo sapiens* (U43746), Pan troglodytes* (XP_509619), Macaca mulatta* (XP_001118184), Rattus Norvegicus* (AAB71378), Canis lupus familiaris* (NP_001006654), Bos Taurus* (XP_583622), Monodelphis domesticus* (EF508680), Gallus gallus (AAL89470), Xenopus laevis (EF508681), Tetraodon nigroviridis (EF564374), Fugu rubripes (not listed), Strongylocentrotus purpuratus (EF523433).

GV (Grantham Variation) is a multiple alignment measure of variation at a given position. A GV=0 means a position is invariant. (GV<31 is indicative of very conservative).
GD (Grantham Deviation) provides a description of the magnitude of sequence variation at its position in a multiple sequence alignment. GD=0 is within the observed range of variation.
Prediction whether the substitution is most likely (Class C65) or least likely (Class C0) to interfere with protein function.
Tavtigian SV, Deffenbaugh AM, Yin L, Judkins T, Scholl T, Samollow PB, de Silva D, Zharkikh A, Thomas A. 2005. Comprehensive statistical study of 452 BRCA1 missense substitutions with classification of eight recurrent substitutions as neutral. J Med Genet 43:295-305.

[7]Classification based on the literature references listed per unclassified variant
BRCA1 p.M18T [5, 10, 12, 15-18]
BRCA1 p.S1655F [1, 2, 4, 7, 9, 19, 21, 23]
BRCA1 p.R1699Q [1, 3, 4, 7, 8, 9, 13, 14, 20, 22, 23]
BRCA2 p.E462G [9, 24]
BRCA2 p.R3052W [6, 9, 11]
BRCA2 p.R3052Q [5, 9, 11]

1. Abkevich, V., Zharkikh, A., Deffenbaugh, A. M., Frank, D., Chen, Y., Shattuck, D., Skolnick, M. H., Gutin, A., and Tavtigian, S. V. Analysis of missense variation in human BRCA1 in the context of interspecific sequence variation. J Med Genet, *41:* 492-507, 2004.

2. Carvalho, M. A., Marsillac, S. M., Karchin, R., Manoukian, S., Grist, S., Swaby, R. F., Urmenyi, T. P., Rondinelli, E., Silva, R., Gayol, L., Baumbach, L., Sutphen, R., Pickard-Brzosowicz, J. L., Nathanson, K. L., Sali, A., Goldgar, D., Couch, F. J., Radice, P., and Monteiro, A. N. Determination of cancer risk associated with germ line BRCA1 missense variants by functional analysis. Cancer Res., *67:* 1494-1501, 2007.

3. Chenevix-Trench, G., Healey, S., Lakhani, S., Waring, P., Cummings, M., Brinkworth, R., Deffenbaugh, A. M., Burbidge, L. A., Pruss, D., Judkins, T., Scholl, T., Bekessy, A., Marsh, A., Lovelock, P., Wong, M., Tesoriero, A., Renard, H., Southey, M., Hopper, J. L., Yannoukakos, K., Brown, M., Easton, D., Tavtigian, S. V., Goldgar, D., and Spurdle, A. B. Genetic and histopathologic evaluation of BRCA1 and BRCA2 DNA sequence variants of unknown clinical significance. Cancer Res., *66:* 2019-2027, 2006.

4. Clapperton, J. A., Manke, I. A., Lowery, D. M., Ho, T., Haire, L. F., Yaffe, M. B., and Smerdon, S. J. Structure and mechanism of BRCA1 BRCT domain recognition of phosphorylated BACH1 with implications for cancer. Nature Structural & Molecular Biology, *11:* 512-518, 2004.

5. Easton, D. F., Deffenbaugh, A. M., Pruss, D., Frye, C., Wenstrup, R. J., len-Brady, K., Tavtigian, S. V., Monteiro, A. N., Iversen, E. S., Couch, F. J., and Goldgar, D. E. A systematic genetic assessment of 1,433 sequence variants of unknown clinical significance in the BRCA1 and BRCA2 breast cancer-predisposition genes. Am. J. Hum. Genet., *81:* 873-883, 2007.

6. Farrugia, D. J., Agarwal, M. K., Pankratz, V. S., Deffenbaugh, A. M., Pruss, D., Frye, C., Wadum, L., Johnson, K., Mentlick, J., Tavtigian, S. V., Goldgar, D. E., and Couch, F. J. Functional assays for classification of BRCA2 variants of uncertain significance. Cancer Res., *68:* 3523-3531, 2008.

7. Glover, J. N. Insights into the Molecular Basis of Human Hereditary Breast Cancer from Studies of the BRCA1 BRCT Domain. Fam. Cancer, *5:* 89-93, 2006.

8. Goldgar, D. E., Easton, D. F., Deffenbaugh, A. M., Monteiro, A. N. A., Tavtigian, S. V., and Couch, F. J. Integrated evaluation of DNA sequence variants of unknown clinical significance: Application to BRCA1 and BRCA2. Am J Hum Genet, *75:* 535-544, 2004.

9. Gómez García EB, Oosterwijk JC, Timmermans M, van Asperen CJ, Hogervorst FB, Hoogerbrugge N, Oldenburg R, Verhoef S, Dommering CJ, Ausems MG, van Os TA, van der Hout AH, Ligtenberg M, van den Ouweland A, van der Luijt RB, Wijnen JT, Gille JJ, Lindsey PJ, Devilee P, Blok MJ, Vreeswijk MP. A method to assess the clinical significance of unclassified variants in the BRCA1 and BRCA2 genes based on cancer family history. Breast Cancer Res. 2009;11:R8.

10. Greenman, J., Mohammed, S., Ellis, D., Watts, S., Scott, G., Izatt, L., Barnes, D., Solomon, E., Hodgson, S., and Mathew, C. Identification of missense and truncating mutations in the BRCA1 gene in sporadic and familial breast and ovarian cancer. Genes Chrom Cancer, *21:* 244-249, 1998.

11. Kutznetsov, S.G., Liu, P., and Sharan, S.k. Mouse embryonic stem cell-based functional assay to evaluate mutations in BRCA2. Nat. Med., *1:* 875-881, 2008

12. Langston, A. A., Malone, K. E., Thompson, J. D., Daling, J. R., and Ostrander, E. A. *BRCA1* mutations in a population-based sample of young women with breast cancer. N. Engl. J. Med., *334:* 137-142, 1996.

13. Lovelock, P. K., Spurdle, A. B., Mok, M. T., Farrugia, D. J., Lakhani, S. R., Healey, S., Arnold, S., Buchanan, D., Investigators, K., Couch, F. J., Henderson, B. R., Goldgar, D. E., Tavtigian, S. V., Chenevix-Trench, G., and Brown, M. A. Identification of BRCA1 missense substitutions that confer partial functional activity: potential moderate risk variants? Breast Cancer Res., *9:* R82, 2007.

14. Mirkovic, N., Marti-Renom, M. A., Weber, B. L., Sali, A., and Monteiro, A. N. A. Structure-based assessment of missense mutations in human BRCA1: Implications for breast and ovarian cancer predisposition. Cancer Res., *64:* 3790-3797, 2004.

15. Morris, J. R., Pangon, L., Boutell, C., Katagiri, T., Keep, N. H., and Solomon, E. Genetic analysis of BRCA1 ubiquitin ligase activity and its relationship to breast cancer susceptibility. Hum. Mol. Genet., *15:* 599-606, 2006.

16. Morris, J. R. and Solomon, E. BRCA1 : BARD1 induces the formation of conjugated ubiquitin structures, dependent on K6 of ubiquitin, in cells during DNA replication and repair. Hum. Mol. Genet., *13:* 807-817, 2004.

17. Ruffner, H., Joazeiro, C. A., Hemmati, D., Hunter, T., and Verma, I. M. Cancer-predisposing mutations within the RING domain of BRCA1: Loss of ubiquitin protein ligase activity and protection from radiation hypersensitivity. Proc. Natl. Acad. Sci. USA, *98:* 5134-5139, 2001.

18. Sarkar, M. and Magliery, T. J. Re-engineering a split-GFP reassembly screen to examine RING-domain interactions between BARD1 and BRCA1 mutants observed in cancer patients. Mol. Biosyst., *4:* 599-605, 2008.

19. Shiozaki, E. N., Gu, L., Yan, N., and Shi, Y. Structure of the BRCT repeats of BRCA1 bound to a BACH1 phosphopeptide: implications for signaling. Mol. Cell, *14:* 405-412, 2004.

20. Vallon-Christersson, J., Cayanan, C., Haraldsson, K., Loman, N., Bergthorsson, J. T., Brondum-Nielsen, K., Gerdes, A. M., Moller, P., Kristoffersson, U., Olsson, H., Borg, A., and Monteiro, A. N. Functional analysis of BRCA1 C-terminal missense mutations identified in breast and ovarian cancer families. Hum. Mol. Genet., *10:* 353-360, 2001.

21. Varma, A. K., Brown, R. S., Birrane, G., and Ladias, J. A. Structural basis for cell cycle checkpoint control by the BRCA1-CtIP complex. Biochemistry, *44:* 10941-10946, 2005.

22. Williams, R. S., Chasman, D. I., Hau, D. D., Hui, B., Lau, A. Y., and Glover, J. N. M. Detection of protein folding defects caused by BRCA1-BRCT truncation and missense mutations. J. Biol. Chem., *278:* 53007-53016, 2003.

23. Williams, R. S., Lee, M. S., Hau, D. D., and Glover, J. N. M. Structural basis of phosphopeptide recognition by the BRCT domain of BRCA1. Nature Structural & Molecular Biology, *11:* 519-525, 2004.

24. Wu, K., Hinson, S. R., Ohashi, A., Farrugia, D., Wendt, P., Tavtigian, S. V., Deffenbaugh, A., Goldgar, D., and Couch, F. J. Functional Evaluation and Cancer Risk Assessment of BRCA2 Unclassified Variants. Cancer Res., *65:* 417-426, 2005.

Table 2: The mean, standard deviation and the r.

	Female, breast cancer	Male, breast cancer	Female, ovarian cancer
Non-carriers			
μ	72	94.5	85
σ	20	20	25
r	0.15	0.0025	0.035
Carriers BRCA1			
μ	53		65.5
σ	16.5		15.5
r	0.96		0.99
Carriers BRCA2			
μ	58.5	58.5	67
σ	13.8	13.8	7.5
r	1	0.15	0.41

Values for mean age of diagnosis (μ), standard deviation (σ) and the life time risk (r) for occurrence of breast- or ovarian cancer are based on the model described in (1).

Table 3: Algorithm 1: Obtaining all possibilities for genotypes of the family members.

```
for generation H = 2, ..., H_max
    for location i = 1, ..., n
        for all existing configurations
            if G_i = H and both parents have G_i = 0
                set G_i = 0
            end
            if G_i = H and one of the parents has G = 1
                split the configuration in two new configurations:
                one with G_i = 0 and one with G_i = 1
            end
        end
    end
end
```

This is the algorithm described in Subsection 2.2

Figure 1: Cumulative rates for breast and ovarian cancer versus age.

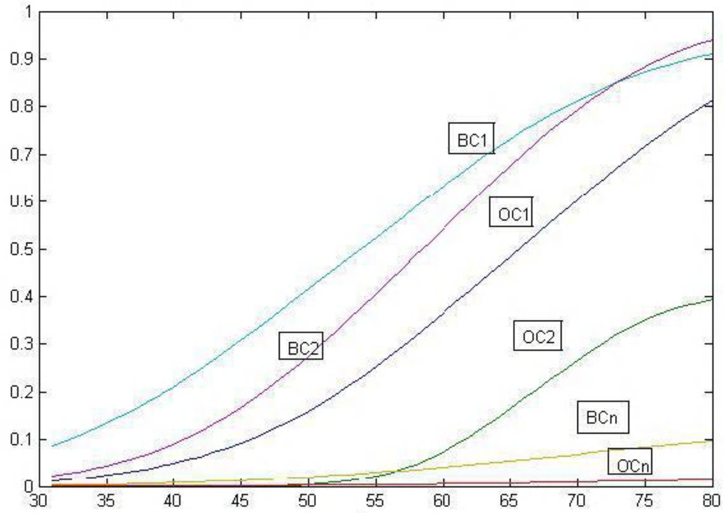

The penetrance of breast and ovarian cancer for general population (BCn/OVn) and for carriers of BRCA1 (BC1/OC1) or BRCA2 (BC2/OC2) mutation is depicted as a function of age (Jonker et al. (1)).

Figure 2: Application of the algorithm of Subsection 2.2

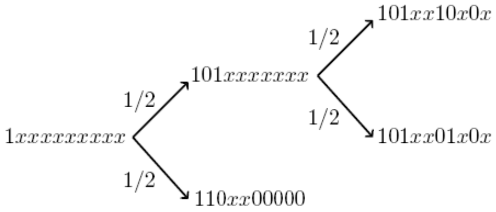

(A)

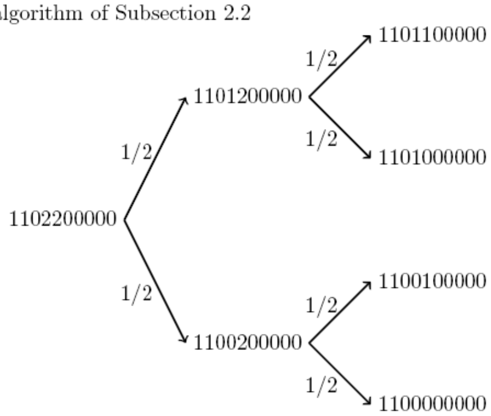

(B)

For description you are referred to Subsection 2.2.

Figure 3:

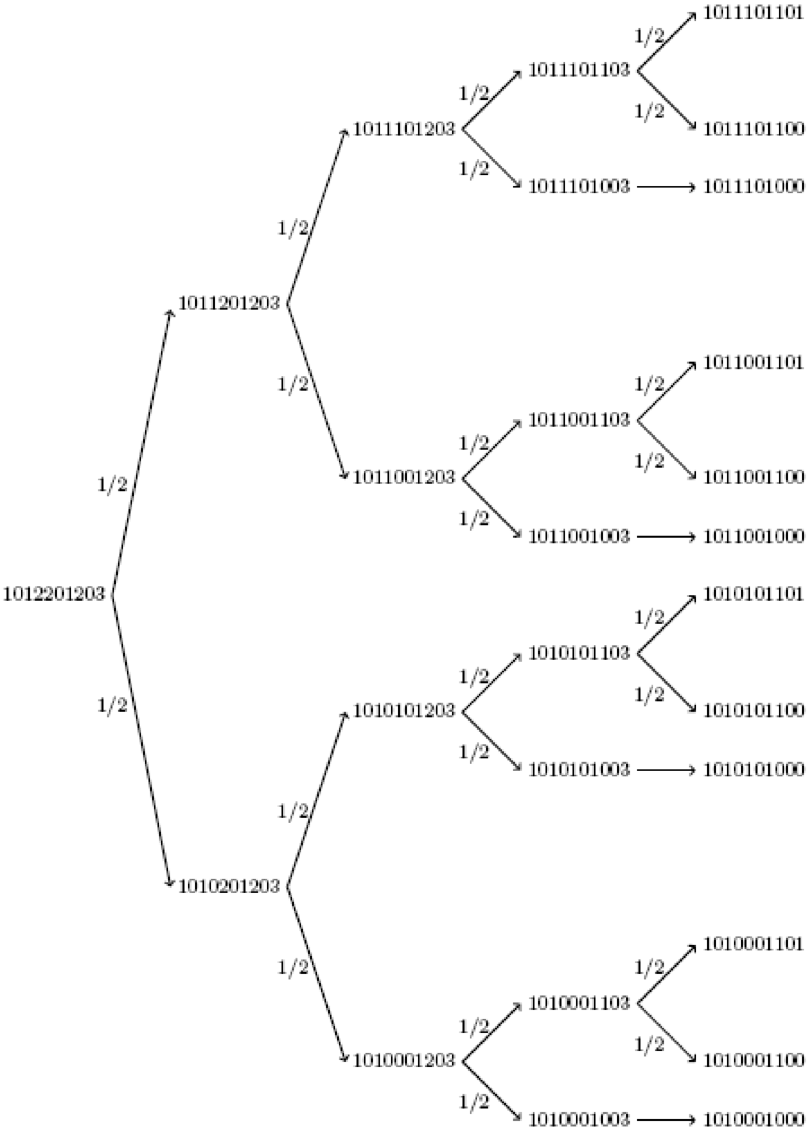

For description you are referred to Subsection 2.2.

Figure 4: Pedigree with variant in BRCA2 (c.135-15_135-12del)

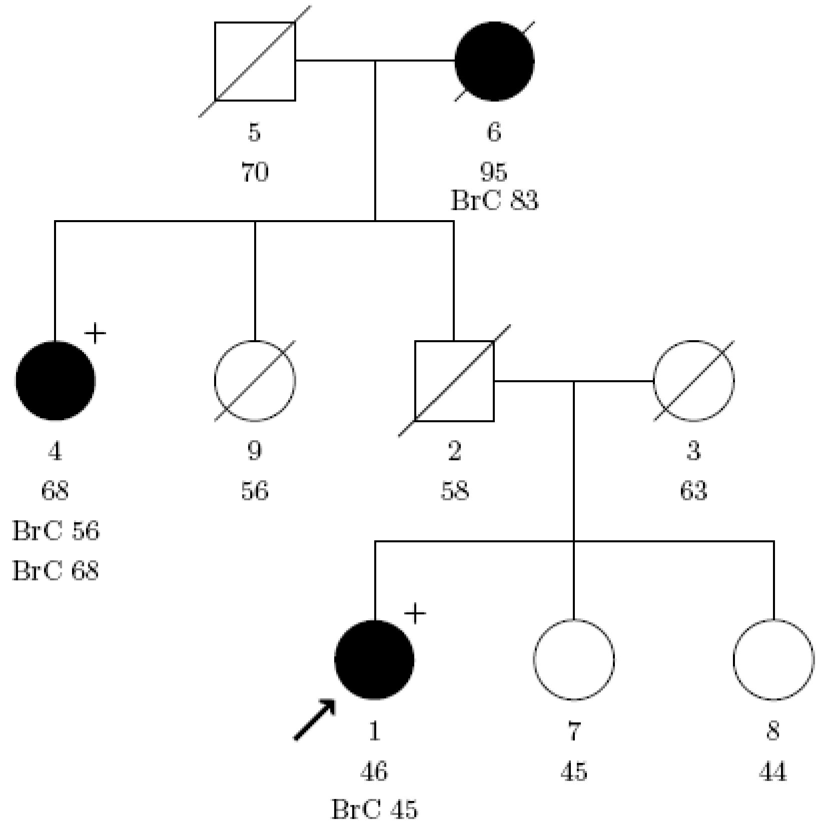

Individuals are numbered (1-9) for identification and below the latest known age is listed.
◯ female, ☐ male, / deceased, ■ affected with breast (or ovarian) cancer at age x (BrC x), in case of bilateral breast cancer this is listed below the first occurrence.
Unless specified by + (carrier of UV), - (no carrier), individuals are not genotyped.

Figure 5:

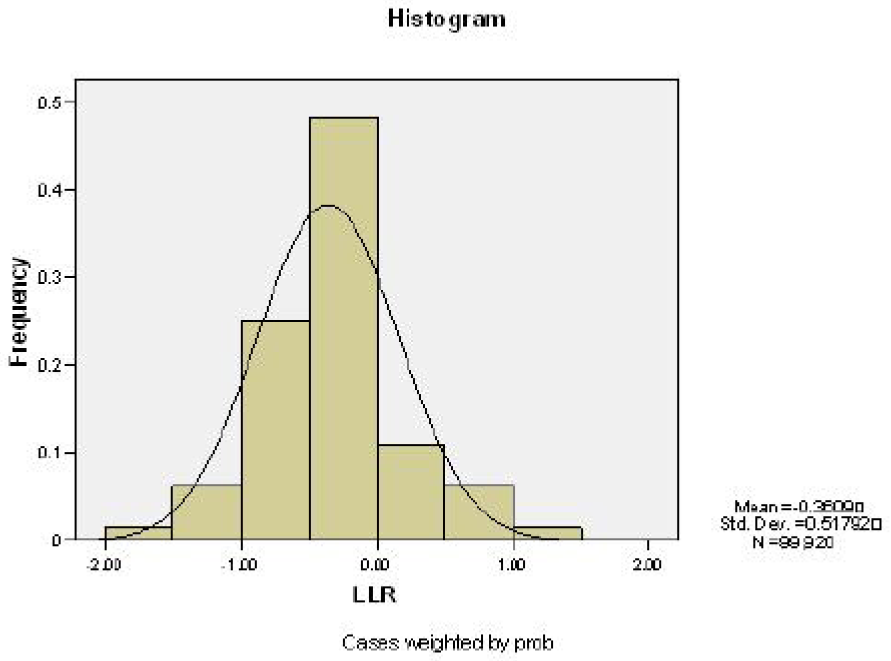

Histogram of the log likelihood ratio for different genotypic patterns of Supplementary Figure 4.
