# Supplementary material for: A simple method for co-segregation analysis to evaluate the pathogenicity of unclassified variants; BRCA1 and BRCA2 as an example
Source: BMC Cancer. 2009 Jun 29;9:211. doi: 10.1186/1471-2407-9-211 (PMC2714556; doi:10.1186/1471-2407-9-211)
Supplement: Additional file 2 — Instruction method Mohammadi. This document contains a description on the use of the co-segregation analysis website with detailed instruction on the preparation of the input file that contains the pedigree information. [file 1471-2407-9-211-S2.pdf]

Instructions co-segregation analysis website

http://www.msbi.nl/cosegregation/

Introduction

We have developed an easy to use method which calculates the likelihood ratio (LR) of an unclassified variant in BRCA1 or BRCA2 to be deleterious. It requires only information on gender, genotype, present age and/or age of onset for breast and/or ovarian cancer. Although co-segregation analysis on itself is in most cases insufficient to prove pathogenicity of an UV, this method simplifies the use of co-segregation as one of the key features in a multifactorial approach considerably.

Input

A table is made based on the pedigree of the family.
Create a table with 10 columns (see below for example).
These columns respectively contain:

1. Person ID.
2. Father ID (0 if there is no father).
3. Mother ID (0 if there is no mother).
4. 1 for proband and 0 for others.
5. Current age (if ages are unknown, estimations can be made for each generation, based on the mean age of this generation).
6. Gender (1=male, 2=female, 9=unknown).
7. Age of onset of the first breast cancer (0 if there is no breast cancer).
8. Age of onset of the second breast cancer (0 if there is no 2nd breast cancer).
9. Age of onset of ovarian cancer (0 if there is no ovarian cancer).
10. Genotype (0 non-carrier, 1 carrier and 2 for unknown genotypes).

Example: Figure 2 from the manuscript.

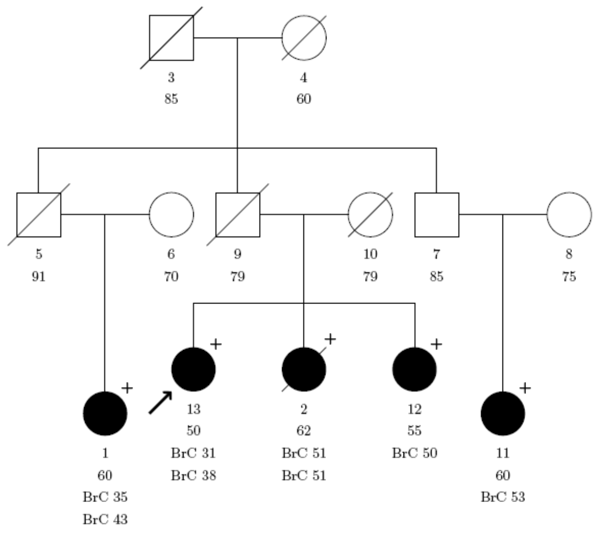

The corresponding matrix

Person ID	Father ID	Mother ID	Proband	Age	Gender	Age of onset BC	Age of onset 2nd BC	Age of onset OC	Genotype
1	5	6	0	60	2	35	43	0	1
2	9	10	0	62	2	51	51	0	1
3	0	0	0	85	1	0	0	0	2
4	0	0	0	60	2	0	0	0	2
5	3	4	0	91	1	0	0	0	2
6	0	0	0	70	2	0	0	0	2
7	3	4	0	85	1	0	0	0	2
8	0	0	0	75	2	0	0	0	2
9	3	3	0	79	1	0	0	0	2
10	0	0	0	79	2	0	0	0	2
11	7	8	0	60	2	53	0	0	1
12	9	10	0	55	2	50	0	0	1
13	9	10	1	50	2	31	38	0	1

Copy the data (not the headings) from the matrix and paste them in simple text format, i.e. notepad.

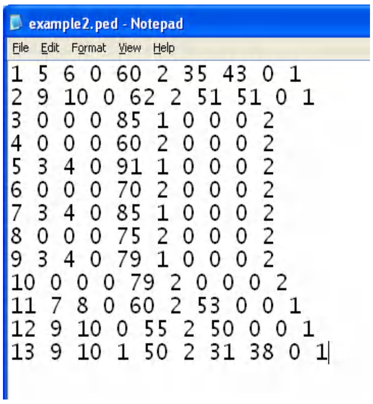
example2.ped - Notepad

File Edit Format View Help

```
1 5 6 0 60 2 35 43 0 1
2 9 10 0 62 2 51 51 0 1
3 0 0 0 85 1 0 0 0 2
4 0 0 0 60 2 0 0 0 2
5 3 4 0 91 1 0 0 0 2
6 0 0 0 70 2 0 0 0 2
7 3 4 0 85 1 0 0 0 2
8 0 0 0 75 2 0 0 0 2
9 3 4 0 79 1 0 0 0 2
10 0 0 0 79 2 0 0 0 2
11 7 8 0 60 2 53 0 0 1
12 9 10 0 55 2 50 0 0 1
13 9 10 1 50 2 31 38 0 1
```

Save the file as follows:

File name*	FamilyID_BRCA1.ped or FamilyID_BRCA2.ped
Save as type	All files
Encoding	ANSI

* Use BRCA1 or BRCA2 depending on the gene in which the variant is located. This is not required but it will simplify the use of the co-segregation analysis program on the website. Due to the difference in penetrance for mutations in BRCA1 and BRCA2, the gene in which the variant is located has to be selected for correct calculation of the likelihood ratio. Description of the gene in the file name will help you to select the gene easily without going back to the family data.

Go to http://www.msbi.nl/cosegregation/

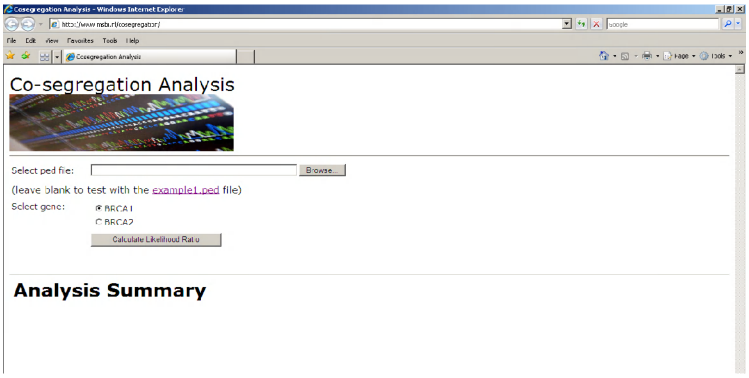

Select a ped file.
Select the gene in which the variant is located.
Calculate Likelihood Ratio.

The Analysis Summary will give the likelihood ratio of the variant being deleterious.
